# Serum ENA-78, CXCL1, and caspase-3 are associated with disease severity and early hearing recovery in idiopathic sudden sensorineural hearing loss

**DOI:** 10.3389/fnins.2026.1901582

**Published:** 2026-08-20

**Authors:** Yang Wang, Xiaodan Zhu, Le Wang, Pengfei Wang, Fanglei Ye, Yulin Zhao

**Affiliations:** 1 Department of Otology, East District, The First Affiliated Hospital of Zhengzhou University, Zhengzhou, Henan, China; 2 Department of Otorhinolaryngology Head and Neck Surgery, The First Affiliated Hospital of Zhengzhou University, Zhengzhou, Henan, China

**Keywords:** apoptosis, biomarker, caspase-3, CXCL1, ENA-78, hearing recovery, idiopathic sudden sensorineural hearing loss, inflammation

## Abstract

**Background:**

Idiopathic sudden sensorineural hearing loss (ISSNHL) is an otologic emergency with highly variable recovery despite prompt treatment. Because inflammatory chemotaxis and apoptosis are implicated in cochlear injury, serum biomarkers reflecting these pathways may improve early risk stratification.

**Methods:**

In this single-center observational study, 209 consecutive adults with unilateral ISSNHL and 209 matched healthy controls were enrolled between July 2024 and February 2026. Patients were stratified by initial pure-tone average into mild, moderate, severe, or profound hearing loss. Fasting serum ENA-78, CXCL1, and caspase-3 were quantified at admission by enzyme-linked immunosorbent assay. After standardized 10-day treatment, complete, marked, or partial hearing improvement was classified as favorable early recovery and ineffective treatment as unfavorable. Analyses included multivariable logistic regression, receiver operating characteristic (ROC) analysis, 1,000-sample bootstrap internal validation, comparison with a clinical base model using net reclassification improvement (NRI) and integrated discrimination improvement (IDI), calibration, decision-curve analysis, a nomogram, and prespecified subgroup analyses.

**Results:**

All three biomarkers were higher in patients than in controls and increased progressively with severity (all *p* < 0.001). After treatment, 138 patients (66.0%) achieved favorable and 71 (34.0%) unfavorable early recovery. The unfavorable group had higher ENA-78 (147.69 ± 33.51 vs. 113.89 ± 21.89 pg/mL), CXCL1 (173.45 ± 36.44 vs. 137.24 ± 25.78 pg/mL), and caspase-3 (9.11 ± 2.43 vs. 6.63 ± 2.18 ng/mL) levels (all *p* < 0.001). Each biomarker remained independently associated with unfavorable recovery (ENA-78: odds ratio [OR], 1.019; CXCL1: OR, 1.024; caspase-3: OR, 1.106). The combined signature showed an apparent area under the curve (AUC) of 0.893 (95% CI, 0.843–0.932; 83.10% sensitivity, 81.88% specificity), which remained high after bootstrap correction (0.879). Adding the biomarkers to the clinical model improved discrimination (AUC, 0.709 to 0.912; NRI, 0.842; IDI, 0.187; all *p* < 0.001), with adequate calibration, positive net benefit across thresholds of 0.12 to 0.72, and consistent performance across subgroups.

**Conclusion:**

Admission serum ENA-78, CXCL1, and caspase-3 were associated with disease severity and early hearing recovery in ISSNHL. Their combined assessment outperformed any single marker and added value beyond routine clinical variables, although multicenter external validation and longer follow-up remain necessary before clinical implementation.

## Introduction

Idiopathic sudden sensorineural hearing loss (ISSNHL) is a sudden decline in sensorineural hearing, usually defined as a loss of at least 30 dB affecting three contiguous frequencies within 72 h, in the absence of an identifiable alternative cause. It is widely regarded as an otologic emergency because early recognition and treatment may influence auditory recovery, yet the clinical course remains remarkably heterogeneous. Some patients experience rapid and substantial hearing improvement, whereas others retain permanent hearing deficits despite apparently appropriate care. This variability complicates prognostic counseling and limits clinicians’ ability to identify, at the time of presentation, those individuals who may require closer monitoring or more aggressive therapeutic strategies (Chandrasekhar et al., 2019; Lee and Chung, 2024; Tsuzuki and Wasano, 2024; Mandavia et al., 2024).

The biological basis of ISSNHL is complex and likely multifactorial. Contemporary evidence supports contributions from cochlear microvascular compromise, endothelial dysfunction, immune-inflammatory activation, oxidative stress, viral or postviral mechanisms, and cochlear cellular injury (Lee and Chung, 2024; Tsuzuki and Wasano, 2024; Mandavia et al., 2024; Wu et al., 2023). The cochlea is especially susceptible to vascular and inflammatory insults because it depends on terminal microcirculation with minimal collateral reserve and has high metabolic demand. Once microvascular perfusion is impaired or local inflammatory activity becomes excessive, a cascade of tissue edema, oxidative damage, barrier dysfunction, and sensory-cell injury may follow (Tsuzuki and Wasano, 2024; Mandavia et al., 2024). This concept has stimulated growing interest in blood-based biomarkers that may capture otherwise occult biological injury and complement routine clinical and audiometric assessment.

Clinical prognostication in ISSNHL has gradually moved beyond simple descriptive analyses. Recent models and nomograms suggest that outcome is shaped by an interplay of baseline pure-tone average, audiogram configuration, age, time from onset to treatment, and vestibular symptoms rather than a single predictor (Mandavia et al., 2024; Wu et al., 2023; Zhang et al., 2023; Shon et al., 2024). Parallel to this trend, several studies have shown that systemic inflammatory indices, including the neutrophil-to-lymphocyte ratio, platelet-to-lymphocyte ratio, systemic immune-inflammation index, and related hematologic markers, are associated with disease occurrence, severity, or hearing recovery (Zhao et al., 2025; Zhang X. et al., 2025; Zhang Q. et al., 2025). Although these markers are accessible, they remain indirect and biologically nonspecific. A more mechanistically focused biomarker approach may better reflect the interplay between inflammatory recruitment and downstream tissue injury in ISSNHL.

Epithelial neutrophil-activating peptide 78 (ENA-78), also known as CXCL5, and C-X-C motif chemokine ligand 1 (CXCL1) are proinflammatory chemokines with strong neutrophil-chemotactic activity. Experimental and translational studies have shown that these mediators participate in leukocyte recruitment, endothelial activation, microvascular obstruction, and amplification of local tissue injury in ischemic or inflammatory settings (Xiao et al., 2022; Huang et al., 2023; Qiao et al., 2024). Such biology is relevant to ISSNHL because cochlear dysfunction may be aggravated by inflammatory microvascular compromise and neutrophil-associated oxidative stress. Caspase-3, in contrast, is a well-established executioner protease in apoptosis. Experimental inner-ear research indicates that inflammatory and oxidative injury can activate cell-death pathways in cochlear hair cells and supporting tissues, which may be associated with a reduced likelihood of meaningful functional recovery (Qiao et al., 2024; Yu et al., 2022; Wu et al., 2025).

Despite increasing interest in biomarker-guided risk assessment, evidence regarding ENA-78, CXCL1, and caspase-3 in ISSNHL remains limited, and prior work has not adequately examined these markers as an integrated inflammatory and apoptosis-associated serum signature. This gap is clinically important because an effective early biomarker panel should do more than distinguish patients from healthy individuals; it should relate to initial hearing-loss severity, identify patients at risk of poor early response, and provide a biologically coherent explanation for why recovery differs among patients who receive comparable treatment. The present study was therefore designed to evaluate admission serum ENA-78, CXCL1, and caspase-3 across three connected levels of clinical relevance: case–control biomarker elevation, severity-dependent biomarker gradients, and early hearing recovery after standardized therapy. We further explored whether combining these markers improves apparent discrimination for unfavorable early recovery compared with single-marker assessment. By linking chemokine-mediated inflammatory recruitment with caspase-3-related apoptosis signaling, this study aims to strengthen the rationale for a mechanistically informed serum biomarker strategy that may support future ISSNHL risk stratification models and guide validation studies in larger multicenter cohorts.

## Methods

### Study design, ethical approval, and participants

This single-center observational study was conducted in the Department of Otology, First Affiliated Hospital of Zhengzhou University, and included consecutive adult patients with unilateral ISSNHL who were hospitalized between July 2024 and February 2026. A contemporaneous healthy control group was recruited from individuals undergoing routine health examination during the same time interval. Controls were matched to patients for age, sex, and body mass index and were additionally screened to exclude conditions capable of altering baseline chemokine or caspase-3 concentrations, as detailed under Eligibility criteria. The study complied with the ethical principles of the Declaration of Helsinki and was approved by the institutional ethics committee of the First Affiliated Hospital of Zhengzhou University. All participants provided written informed consent before enrollment. Reporting was structured according to the principles of the STROBE statement for observational studies and the TRIPOD+AI framework for prediction-oriented analyses (Collins et al., 2024; Wolff et al., 2019; von Elm et al., 2007). The completed STROBE and TRIPOD+AI checklists, together with the supplementary statistical tables cited in the Results, are provided as Supplementary materials.

**Figure 1:**
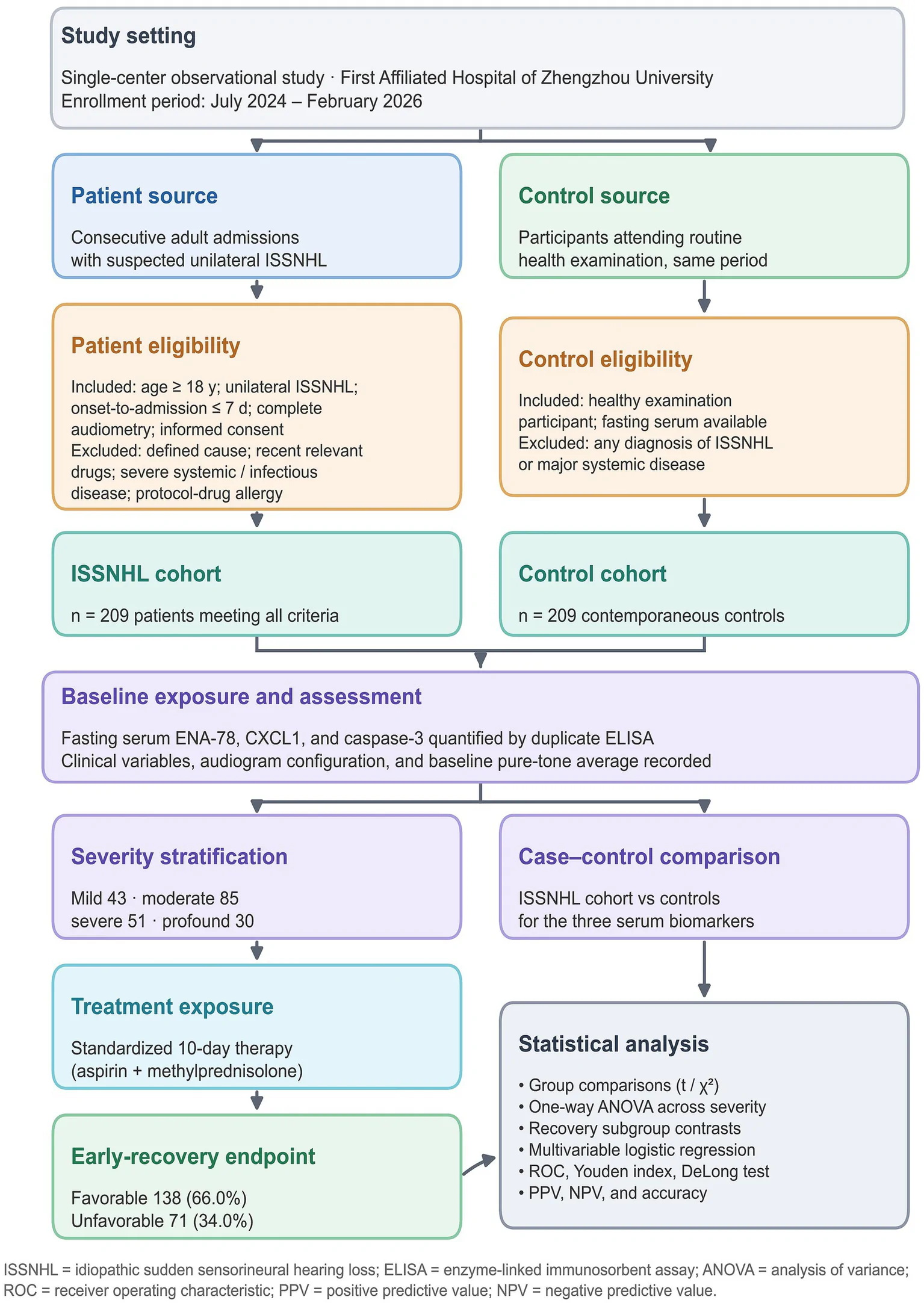
STROBE-adapted cohort flow diagram and analytic framework. The figure summarizes patient and control recruitment, eligibility application, biomarker measurement, severity stratification, treatment exposure, endpoint formation, and the main analytic steps used in the study.

ISSNHL was defined in accordance with accepted diagnostic criteria as hearing loss occurring within 72 h, involving at least three contiguous frequencies, and not attributable to another identified cause after clinical evaluation (Chandrasekhar et al., 2019; Editorial Board of Chinese Journal of Otorhinolaryngology Head and Neck Surgery, Society of Otorhinolaryngology Head and Neck Surgery, Chinese Medical Association, 2015). The overall study flow, including patient enrollment, control recruitment, biomarker assessment, severity classification, treatment, and outcome analysis, is shown in Figure 1.

### Eligibility criteria

Inclusion criteria:

- Age 18 years or older.
- Unilateral ISSNHL diagnosed according to guideline-based criteria.
- Interval from symptom onset to hospital admission of 7 days or less.
- Complete baseline and posttreatment pure-tone audiometric assessment.
- Provision of written informed consent.

Exclusion criteria:

- Hearing loss attributable to congenital inner-ear malformation, Ménière disease, otitis media, autoimmune inner-ear disease, ear trauma, vestibular schwannoma, or another clearly defined cause.
- Use of immunosuppressive agents, systemic glucocorticoids, or anticoagulants within 1 month before enrollment.
- Severe malignancy or major cardiac, hepatic, renal, or hematologic disease.
- Acute or chronic infectious disease at the time of enrollment.
- Known allergy to protocol medications or inability to complete the required audiometric follow-up.

Additional exclusion criteria for healthy controls:

- Any allergic disorder, autoimmune disease, or vasculitis.
- Acute or chronic infectious disease.
- Use of anti-inflammatory drugs or systemic glucocorticoids within 1 month before blood sampling.
- Any prior or current diagnosis of ISSNHL.
- Major cardiac, hepatic, renal, or hematologic disease.

### Clinical assessment and severity classification

Clinical data were recorded on a standardized case-report form by trained investigators who had undergone protocol-specific instruction before the start of data collection. Variables included age, sex, body mass index (BMI), diabetes mellitus, hypertension, hyperlipidemia, affected side, time from symptom onset to hospital admission, and audiogram configuration. Baseline pure-tone audiometry was performed after admission. The mean hearing threshold at 500, 1,000, 2,000, and 4,000 Hz (the pure-tone average, PTA) was used to quantify initial hearing loss and to classify disease severity as mild, moderate, severe, or profound according to the study protocol and the referenced national guideline (Editorial Board of Chinese Journal of Otorhinolaryngology Head and Neck Surgery, Society of Otorhinolaryngology Head and Neck Surgery, Chinese Medical Association, 2015). Audiogram configuration was categorized as total deafness, low-frequency type, high-frequency type, or flat type. To ensure diagnostic consistency, all enrolled cases were independently reviewed by at least two attending otologists.

### Serum biomarker measurement

For control participants, fasting venous blood was collected on the day of routine physical examination. For patients with ISSNHL, fasting venous blood was obtained in the morning after admission and before completion of the treatment course. Three milliliters of venous blood were collected, centrifuged at 3,000 r/min for 15 min with an 8-cm centrifugation radius, and the serum was separated, aliquoted, and stored at −80 °C until analysis.

Serum ENA-78, CXCL1, and caspase-3 concentrations were measured by enzyme-linked immunosorbent assay using commercial Thermo Fisher Scientific kits according to the manufacturer instructions. Before analysis, samples were thawed at room temperature and centrifuged again at 3,000 r/min for 5 min to remove sediment. Standards, blanks, and serum samples were loaded at 100 μL per well, incubated at room temperature for 2 h, and subsequently exposed to biotinylated antibody working solution for 1 h. After five wash cycles, enzyme conjugate was added and incubated for 30 min protected from light. A chromogenic substrate solution was then applied for 15 min, followed by the stopping solution. Optical density was measured within 15 min at 450 nm using a Multiskan FC microplate reader. Standard curves were generated for each assay, and concentrations were calculated from the observed optical density values. All samples were analyzed in duplicate and the mean value was used for analysis. Assays were performed by the same experienced laboratory technician under standardized conditions, and the intra-assay and inter-assay coefficients of variation were both maintained below 10%.

### Treatment protocol and outcome definition

All patients received standardized treatment according to the departmental protocol derived from guideline-based care (Editorial Board of Chinese Journal of Otorhinolaryngology Head and Neck Surgery, Society of Otorhinolaryngology Head and Neck Surgery, Chinese Medical Association, 2015). The regimen consisted of oral aspirin enteric-coated tablets at a dose of 75 mg once daily combined with intravenous methylprednisolone sodium succinate at 0.8 mg/kg per day, not exceeding 60 mg daily, diluted in 120 mL of 5% glucose injection. Treatment was continued for 10 consecutive days.

At the end of treatment, repeat pure-tone audiometry was performed in the affected ear by the same audiology technician, who was unaware of the serum biomarker results. Therapeutic response was categorized into four levels according to guideline-based criteria: complete recovery, defined as restoration of the affected frequencies to the normal level or to the pre-illness or contralateral-ear level; marked recovery, defined as a mean hearing improvement of at least 30 dB at the affected frequencies; partial recovery, defined as an improvement of 15 to 30 dB; and no recovery, defined as improvement of less than 15 dB. For prognostic analysis, complete, marked, and partial recovery were grouped as favorable early recovery, whereas no recovery was grouped as unfavorable early recovery.

### Quality control and statistical analysis

Several measures were implemented to improve data quality and analytic consistency. Investigators were trained before data collection, eligibility was verified by two senior clinicians, and data entry was performed independently by two researchers with subsequent cross-checking. Laboratory specimens were coded and tested in duplicate under blinded conditions, and the audiology technician who evaluated posttreatment hearing thresholds was unaware of group allocation and biomarker findings.

Potential sources of bias were considered at each stage and were mitigated as far as the design allowed. Selection bias was limited by enrolling consecutive eligible admissions rather than a convenience sample and by recruiting contemporaneous controls over the same interval. Measurement bias was reduced by quantifying every biomarker in duplicate on a single validated ELISA platform under standardized conditions, with intra-assay and inter-assay coefficients of variation below 10%. Observer bias was minimized because the audiologist who performed posttreatment audiometry was blinded to both group allocation and biomarker results, and laboratory personnel were unaware of clinical outcome. Residual confounding cannot be excluded, and its potential influence is considered in the Discussion.

Statistical analysis was performed with SPSS version 26.0. Continuous variables were assessed for approximate normality and are presented as mean±SD; categorical variables are presented as number (percentage). Between-group comparisons involving two independent groups were performed with the independent-samples *t* test or the χ^2^ test as appropriate. Comparisons across the four severity categories were performed using one-way analysis of variance, followed by Bonferroni-corrected pairwise *post hoc* comparisons between adjacent severity strata. Variables associated with early recovery at *p* < 0.05 in univariable comparisons were entered into a multivariable logistic regression model to identify factors independently associated with unfavorable early recovery. Multicollinearity was assessed before model entry; a variance inflation factor of 5 or greater was prespecified as evidence of problematic collinearity. In the logistic model, unfavorable early recovery was coded as 1 and favorable early recovery as 0. Regression coefficients, standard errors, Wald χ^2^ statistics, ORs, and 95% CIs were reported. To improve interpretability, effect estimates were also summarized using clinically interpretable increments where appropriate. The predictive performance of each biomarker and of the combined serum signature was evaluated with receiver operating characteristic (ROC) analysis. The optimal cutoff for each model was determined by maximizing the Youden index. Sensitivity, specificity, positive predictive value, negative predictive value, and overall accuracy were calculated using the observed prevalence of unfavorable early recovery in the analytic cohort. Area under the curve (AUC) values were compared by DeLong testing. Two-sided *p* values less than 0.05 were considered statistically significant.

The normality of continuous variables was examined with the Shapiro–Wilk test together with inspection of histograms and normal quantile-quantile plots before parametric methods were applied. The dataset was complete for all prespecified clinical, audiometric, and biomarker variables, so that no imputation was required.

Prediction modeling followed a prespecified sequence. A clinical base model was first constructed from the routinely available prognostic variables in the cohort, namely age, sex, BMI, diabetes mellitus, hypertension, hyperlipidemia, onset-to-admission interval, baseline PTA, and audiogram configuration. The three serum biomarkers were then added to this base model to form a combined clinical-plus-biomarker model, and a biomarker-only model was retained for comparison. The incremental contribution of the biomarkers was quantified as the change in the AUC between the base and combined models, tested with the DeLong method, and was further assessed with the continuous net reclassification improvement (NRI) and the integrated discrimination improvement (IDI), each reported with bootstrap confidence intervals.

Model calibration was evaluated with the Hosmer-Lemeshow goodness-of-fit test and the calibration slope and was displayed as a calibration curve relating predicted probabilities to observed early-recovery frequencies. For bedside interpretation, a nomogram was derived from the combined biomarker model so that an individual predicted probability of unfavorable early recovery could be read directly from the admission biomarker values.

To address the optimism inherent in apparent performance, internal validation was performed with 1,000-sample bootstrap resampling. For each model the apparent AUC, the mean optimism, and the resulting bootstrap-corrected AUC were reported together with corrected sensitivity and specificity, and the corrected values were compared with the uncorrected estimates.

Clinical usefulness was examined with decision-curve analysis (DCA), which plots the net benefit of the combined model, the clinical base model, and the default treat-all and treat-none strategies across the plausible range of threshold probabilities, and the threshold range over which the biomarker model yielded positive net benefit was recorded.

Finally, prespecified subgroup analyses examined the stability of biomarker discrimination across clinically relevant strata. Patients were stratified by audiogram configuration, by onset-to-admission interval (3 days or less versus 4 to 7 days), and by baseline severity (mild or moderate versus severe or profound), and the AUC of the combined signature was calculated within each subgroup. Bootstrap resampling, NRI and IDI estimation, calibration, decision-curve analysis, and subgroup modeling were conducted in R version 4.3.1, and all remaining analyses were performed in SPSS version 26.0.

No *a priori* sample-size calculation was performed, because the study enrolled all consecutive eligible patients admitted during a fixed 18-month period rather than a predetermined target. A *post hoc* estimate indicated adequate statistical power. The early-recovery contrasts in the three biomarkers all corresponded to a Cohen d above 1.0, for which the 71 patients with unfavorable recovery and the 138 with favorable recovery provided greater than 99% power at a two-sided *α* of 0.05, and the 71 outcome events supported a three-predictor multivariable model while remaining within accepted limits for events per variable.

## Results

### Study population and baseline characteristics

A total of 209 adults with unilateral ISSNHL met the study criteria and were included in the analytic cohort, together with 209 healthy controls. Within the ISSNHL cohort, 43 patients (20.6%) were classified as having mild disease, 85 (40.7%) moderate disease, 51 (24.4%) severe disease, and 30 (14.4%) profound disease according to the baseline pure-tone average. Audiogram configuration was distributed as total deafness in 38 patients (18.2%), low-frequency type in 52 (24.9%), high-frequency type in 49 (23.4%), and flat type in 70 (33.5%). The affected ear was the right ear in 117 patients (56.0%) and the left ear in 92 (44.0%). The mean interval from symptom onset to hospital admission was 3.21 ± 1.09 days.

Compared with controls, patients with ISSNHL were similar in BMI, age, and sex distribution, indicating that the control group was broadly comparable with respect to these basic demographic characteristics. Mean BMI was 23.12 ± 1.56 kg/m^2^ in the ISSNHL group and 22.96 ± 1.48 kg/m^2^ in the control group (*p* = 0.283). Mean age was 53.12 ± 4.48 years and 52.89 ± 4.33 years, respectively (*p* = 0.594). Male participants accounted for 53.59% of the ISSNHL group and 52.15% of the control group (*p* = 0.769). The baseline comparison is summarized in Table 1, while the internal clinical profile of the ISSNHL cohort is presented in Table 2 and Figure 1.

**Table 1:** Baseline demographic comparison and serum biomarker differences between patients with ISSNHL and healthy controls.

Variable	ISSNHL group (*n* = 209)	Control group (*n* = 209)	Mean difference or contrast	Effect size	Statistic	*p* value
BMI, kg/m^2^	23.12 ± 1.56	22.96 ± 1.48	0.16	Cohen d = 0.11	*t* = 1.076	0.283
Age, years	53.12 ± 4.48	52.89 ± 4.33	0.23	Cohen d = 0.05	*t* = 0.534	0.594
Male sex, *n* (%)	112 (53.59)	109 (52.15)	1.44 percentage points	—	χ^2^ = 0.086	0.769
Female sex, *n* (%)	97 (46.41)	100 (47.85)	−1.44 percentage points	—		
ENA-78, pg/mL	125.37 ± 24.61	62.34 ± 5.38	63.03 (101.1% higher)	Cohen d = 3.54	*t* = 36.172	<0.001
CXCL1, pg/mL	149.54 ± 33.25	74.77 ± 6.23	74.77 (100.0% higher)	Cohen d = 3.13	*t* = 31.953	<0.001
Caspase-3, ng/mL	7.47 ± 2.12	3.32 ± 1.07	4.15 (125.0% higher)	Cohen d = 2.47	*t* = 25.264	<0.001

**Table 2:** Clinical and audiologic profile of the ISSNHL analytic cohort.

Domain	Characteristic	Value	Interpretive focus
Comorbidities	Diabetes mellitus, *n* (%)	15 (7.18)	Vascular-metabolic background
Comorbidities	Hypertension, *n* (%)	31 (14.83)	Vascular risk profile
Comorbidities	Hyperlipidemia, *n* (%)	22 (10.53)	Vascular risk profile
Audiogram configuration	Total deafness type, *n* (%)	38 (18.18)	Audiologic phenotype
Audiogram configuration	Low-frequency type, *n* (%)	52 (24.88)	Audiologic phenotype
Audiogram configuration	High-frequency type, *n* (%)	49 (23.44)	Audiologic phenotype
Audiogram configuration	Flat type, *n* (%)	70 (33.49)	Most frequent pattern
Affected side	Right ear, *n* (%)	117 (55.98)	Laterality
Affected side	Left ear, *n* (%)	92 (44.02)	Laterality
Timing	Onset-to-admission interval, days	3.21 ± 1.09	Early treatment window
Initial severity	Mild, *n* (%)	43 (20.57)	Baseline PTA category
Initial severity	Moderate, *n* (%)	85 (40.67)	Largest severity group
Initial severity	Severe, *n* (%)	51 (24.40)	Baseline PTA category
Initial severity	Profound, *n* (%)	30 (14.35)	Highest initial severity
Early recovery	Favorable, *n* (%)	138 (66.03)	Complete, marked, or partial recovery
Early recovery	Unfavorable, *n* (%)	71 (33.97)	Ineffective response

### Case-control differences in serum ENA-78, CXCL1, and caspase-3

**Figure 2:**
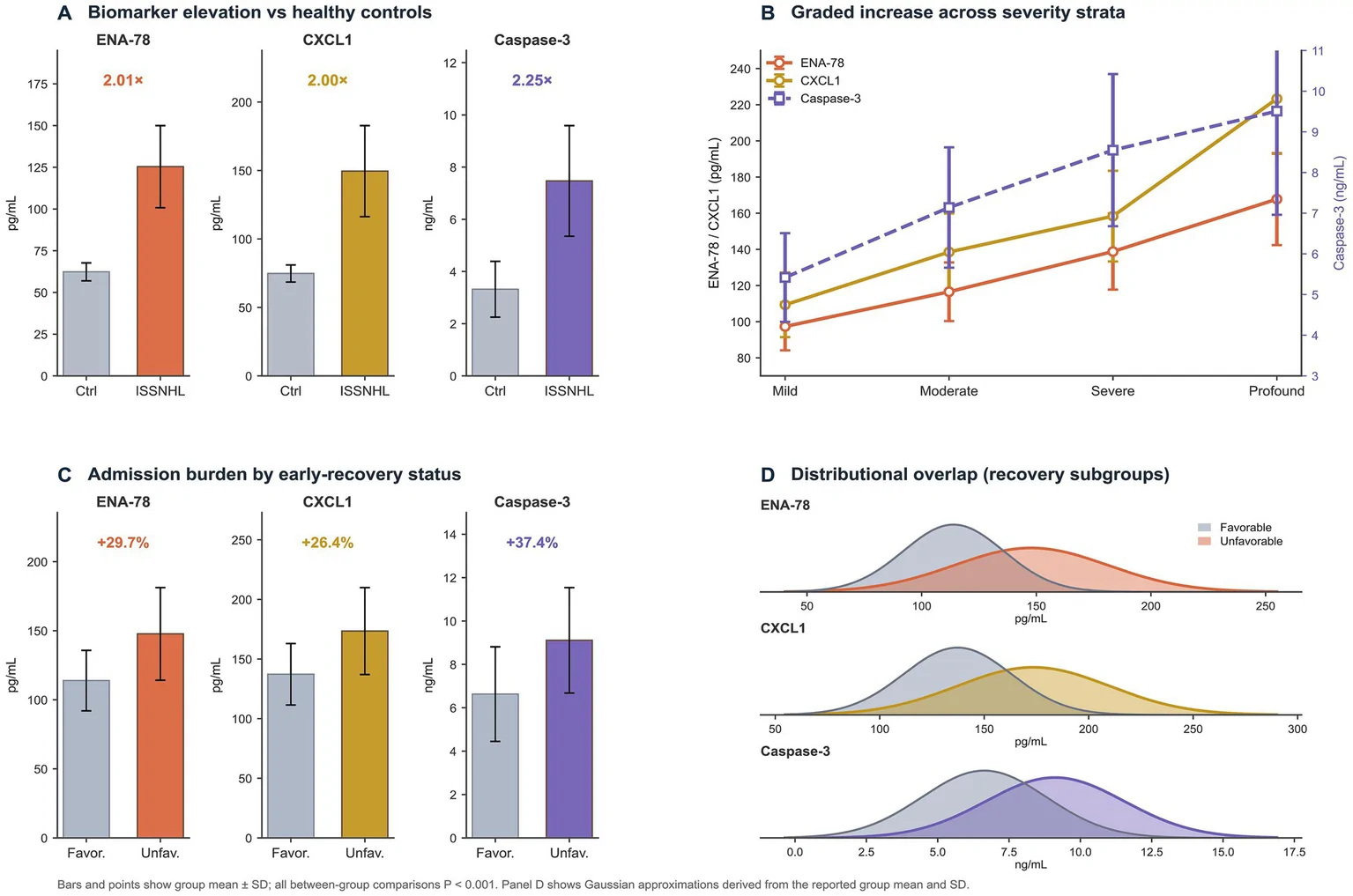
Quantitative biomarker landscape across case status, severity, and early recovery. **(A)** Shows the magnitude of biomarker elevation in patients with ISSNHL relative to healthy controls (mean ± SD), with the patient-to-control fold change annotated. **(B)** Illustrates the graded, monotonic increase in absolute biomarker concentrations (mean ± SD) across the four severity categories, from mild to profound hearing loss. **(C)** Compares the admission biomarker burden between favorable and unfavorable early-recovery subgroups (mean ± SD), with the relative between-group increase annotated. **(D)** Shows the modeled distributional overlap between the two recovery subgroups for each biomarker (Gaussian approximation derived from the reported group mean and SD), illustrating the separation that underlies the discrimination quantified in Figure 3. Units are pg/mL for ENA-78 and CXCL1 and ng/mL for caspase-3.

All three biomarkers were markedly elevated in the ISSNHL cohort relative to healthy controls. Mean serum ENA-78 was 125.37 ± 24.61 pg/mL in patients and 62.34 ± 5.38 pg/mL in controls, corresponding to an approximate doubling of the mean concentration and a highly significant between-group difference (t = 36.172, *p* < 0.001). Mean serum CXCL1 was likewise substantially higher in patients than in controls (149.54 ± 33.25 vs. 74.77 ± 6.23 pg/mL; t = 31.953, *p* < 0.001). Caspase-3 showed the largest relative increase, with mean levels of 7.47 ± 2.12 ng/mL in patients and 3.32 ± 1.07 ng/mL in controls (t = 25.264, p < 0.001). Table 1 reports absolute mean differences, relative increases, and standardized effect sizes, while Figure 2A visualizes the magnitude of separation between patients and controls.

### Relationship between biomarker levels and initial hearing-loss severity

A graded association was observed between biomarker concentration and disease severity. ENA-78 rose progressively from 97.33 ± 13.21 pg/mL in mild ISSNHL to 116.54 ± 16.22 pg/mL in moderate disease, 138.79 ± 21.05 pg/mL in severe disease, and 167.77 ± 25.42 pg/mL in profound disease. The overall between-group difference was significant (*F* = 100.624, *p* < 0.001), and *post hoc* testing showed that each successive severity category had higher ENA-78 levels than the preceding category.

A similar pattern was seen for CXCL1. Mean CXCL1 increased from 109.33 ± 17.89 pg/mL in mild disease to 138.54 ± 21.29 pg/mL in moderate disease, 158.37 ± 25.08 pg/mL in severe disease, and 223.33 ± 30.55 pg/mL in profound disease (*F* = 153.477, *p* < 0.001). The steepest increment was observed between severe and profound disease, suggesting particularly intense chemokine activation in the most severe presentations.

Caspase-3 also increased steadily across severity strata, with mean values of 5.42 ± 1.09 ng/mL in mild disease, 7.14 ± 1.48 ng/mL in moderate disease, 8.55 ± 1.87 ng/mL in severe disease, and 9.51 ± 2.55 ng/mL in profound disease (*F* = 42.820, *p* < 0.001). Table 3 presents the absolute levels, changes from the mild group, and fold enrichment across severity strata. The coordinated severity trajectory of the three biomarkers is visualized in Figure 2B.

**Table 3:** Serum ENA-78, CXCL1, and caspase-3 levels according to initial hearing-loss severity.

Severity group	*n*	ENA-78, pg/mL	Change from mild	CXCL1, pg/mL	Change from mild	Caspase-3, ng/mL	Change from mild
Mild	43	97.33 ± 13.21	Reference	109.33 ± 17.89	Reference	5.42 ± 1.09	Reference
Moderate	85	116.54 ± 16.22	+19.21; 1.20x	138.54 ± 21.29	+29.21; 1.27x	7.14 ± 1.48	+1.72; 1.32x
Severe	51	138.79 ± 21.05	+41.46; 1.43x	158.37 ± 25.08	+49.04; 1.45x	8.55 ± 1.87	+3.13; 1.58x
Profound	30	167.77 ± 25.42	+70.44; 1.72x	223.33 ± 30.55	+114.00; 2.04x	9.51 ± 2.55	+4.09; 1.75x
Overall test		F = 100.624	P < 0.001	F = 153.477	P < 0.001	F = 42.820	P < 0.001

*Post hoc* note: Moderate > mild; severe > moderate; profound > severe for all biomarkers.

### Early hearing recovery and subgroup comparisons

After completion of the standardized 10-day treatment regimen, 138 patients (66.0%) met the criteria for favorable early recovery, whereas 71 patients (34.0%) were classified as having unfavorable early recovery. Basic demographic and clinical characteristics available in the dataset did not differ significantly between these recovery groups. Specifically, BMI, age, sex, comorbid diabetes, hypertension, hyperlipidemia, audiogram type, affected side, and onset-to-admission interval were comparable between groups (all *p* > 0.05), indicating that the observed outcome differences were not explained by these measured baseline variables alone.

In contrast, all three serum biomarkers differed substantially according to early recovery status. Patients with unfavorable early recovery had a mean ENA-78 level of 147.69 ± 33.51 pg/mL compared with 113.89 ± 21.89 pg/mL in those with favorable recovery (t = 8.767, *p* < 0.001), an absolute difference of 33.80 pg/mL and a relative increase of 29.7%. Mean CXCL1 levels were 173.45 ± 36.44 pg/mL in the unfavorable recovery group and 137.24 ± 25.78 pg/mL in the favorable recovery group (t = 8.316, *p* < 0.001), corresponding to a 36.21 pg/mL absolute difference and a 26.4% relative increase. Mean caspase-3 levels were 9.11 ± 2.43 ng/mL and 6.63 ± 2.18 ng/mL, respectively (t = 7.488, *p* < 0.001), corresponding to a 2.48 ng/mL absolute difference and a 37.4% relative increase. These between-group contrasts indicate that patients who did not respond adequately to initial therapy entered treatment with a substantially heavier inflammatory-apoptotic biomarker burden. The subgroup comparisons are summarized in Table 4 and visually highlighted in Figures 2C,D.

**Table 4:** Clinical features and serum biomarker levels according to early hearing recovery status.

Characteristic	Unfavorable early recovery (*n* = 71)	Favorable early recovery (*n* = 138)	Absolute difference or contrast	Effect size	Statistic	*p* value
BMI, kg/m^2^	23.18 ± 1.61	23.09 ± 1.58	0.09	Cohen d = 0.06	*t* = 0.388	0.699
Age, years	53.25 ± 4.53	53.05 ± 4.47	0.20	Cohen d = 0.04	*t* = 0.305	0.761
Male sex, *n* (%)	37 (52.11)	75 (54.35)	−2.24 percentage points	—	χ^2^ = 0.094	0.759
Diabetes mellitus, *n* (%)	7 (9.86)	8 (5.80)	4.06 percentage points	—	χ^2^ = 1.161	0.281
Hypertension, *n* (%)	12 (16.90)	19 (13.77)	3.13 percentage points	—	χ^2^ = 0.364	0.546
Hyperlipidemia, *n* (%)	8 (11.27)	14 (10.14)	1.13 percentage points	—	χ^2^ = 0.063	0.802
Audiogram type distribution	Total 14; low 17; high 16; flat 24	Total 24; low 35; high 33; flat 46	No significant distributional difference	—	χ^2^ = 0.219	0.975
Affected side distribution	Right 39; left 32	Right 78; left 60	No significant laterality difference	—	χ^2^ = 0.048	0.826
Onset-to-admission interval, days	3.32 ± 1.07	3.15 ± 1.06	0.17	Cohen d = 0.16	*t* = 1.095	0.275
ENA-78, pg/mL	147.69 ± 33.51	113.89 ± 21.89	33.80 (29.7% higher)	Cohen d = 1.28	*t* = 8.767	<0.001
CXCL1, pg/mL	173.45 ± 36.44	137.24 ± 25.78	36.21 (26.4% higher)	Cohen d = 1.21	*t* = 8.316	<0.001
Caspase-3, ng/mL	9.11 ± 2.43	6.63 ± 2.18	2.48 (37.4% higher)	Cohen d = 1.09	*t* = 7.488	<0.001

### Multivariable logistic regression analysis

To clarify whether the three biomarkers retained independent associations with outcome when considered together, a multivariable logistic regression model was constructed with early recovery status as the dependent variable. Before model fitting, collinearity diagnostics showed variance inflation factors below the prespecified threshold of 5 for all entered variables, indicating that the model was not adversely affected by problematic multicollinearity.

**Figure 3:**
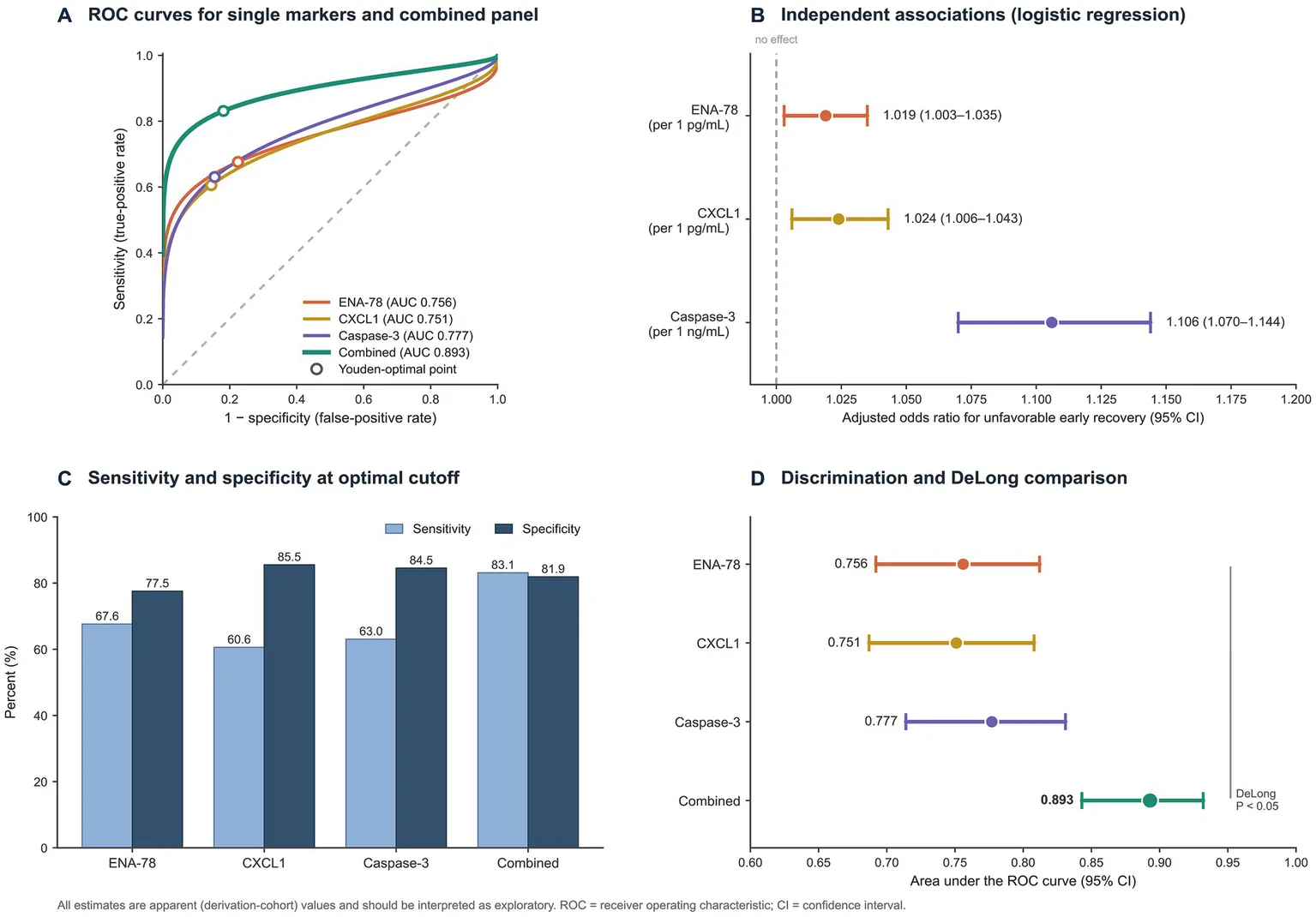
Predictive performance dashboard for the serum biomarker panel. **(A)** Shows the receiver operating characteristic (ROC) curves for each single biomarker and for the combined serum signature, with areas under the curve given in the legend, the Youden-optimal operating point marked on each curve, and the diagonal reference line indicating chance discrimination. Panel **(B)** is a forest plot of the adjusted odds ratios (with 95% CIs) for unfavorable early recovery from the multivariable logistic regression model, referenced to the line of no effect at an odds ratio of 1. **(C)** Compares sensitivity and specificity at the Youden-optimal cutoff for each model. **(D)** Displays the areas under the curve with 95% CIs and indicates the DeLong comparisons showing that the combined signature significantly outperformed each individual biomarker (*p* < 0.05). All performance estimates are apparent values from the derivation cohort and should be interpreted as exploratory.

In the final model, each biomarker remained significantly associated with unfavorable early recovery. For ENA-78, the regression coefficient was 0.019 with a standard error of 0.008, corresponding to an OR of 1.019 (95% CI, 1.003–1.035; *p* = 0.018) for each 1 pg/mL increase. For CXCL1, the regression coefficient was 0.024 with a standard error of 0.009, yielding an OR of 1.024 (95% CI, 1.006–1.043; *p* = 0.008). Caspase-3 showed the strongest association, with a regression coefficient of 0.101, a standard error of 0.017, and an OR of 1.106 (95% CI, 1.070–1.144; *p* < 0.001). Table 5 additionally provides interpretable effect estimates per 10 pg/mL increase for ENA-78 and CXCL1 and per 1 ng/mL increase for caspase-3. The regression findings are visually summarized in the forest plot in Figure 3B.

**Table 5:** Multivariable logistic regression for unfavorable early hearing recovery.

Variable	*β*	SE	Wald χ^2^	*p* value	OR per model unit (95% CI)	Interpretable OR (95% CI)	Interpretation
ENA-78	0.019	0.008	5.641	0.018	1.019 (1.003–1.035)	1.207 (1.030–1.411) per 10 pg/mL	Higher level associated with unfavorable early recovery
CXCL1	0.024	0.009	7.111	0.008	1.024 (1.006–1.043)	1.268 (1.062–1.524) per 10 pg/mL	Higher level associated with unfavorable early recovery
Caspase-3	0.101	0.017	35.298	<0.001	1.106 (1.070–1.144)	1.106 (1.070–1.144) per 1 ng/mL	Higher level associated with unfavorable early recovery

### Exploratory predictive performance of single biomarkers and the combined serum signature

ROC analysis was used to evaluate the discriminative capacity of each biomarker separately and of the combined serum signature derived from the logistic model. Each single biomarker showed moderate apparent ability to distinguish patients with unfavorable early recovery from those with favorable recovery. ENA-78 yielded an AUC of 0.756 (95% CI, 0.692–0.812) with an optimal cutoff of 132.00 pg/mL, 67.61% sensitivity, 77.54% specificity, and a Youden index of 0.451. CXCL1 yielded an AUC of 0.751 (95% CI, 0.687–0.808) with an optimal cutoff of 164.64 pg/mL, 60.56% sensitivity, 85.51% specificity, and a Youden index of 0.461. Caspase-3 performed slightly better than the two chemokines when evaluated individually, with an AUC of 0.777 (95% CI, 0.714–0.831), a cutoff of 7.10 ng/mL, 63.04% sensitivity, 84.51% specificity, and a Youden index of 0.476.

The combined serum signature showed substantially stronger apparent discriminative performance than any single biomarker. Its AUC was 0.893 (95% CI, 0.843–0.932), with 83.10% sensitivity, 81.88% specificity, and a Youden index of 0.650. Based on the observed prevalence of unfavorable early recovery in the cohort, the combined signature corresponded to an estimated positive predictive value of 70.2%, negative predictive value of 90.4%, and overall accuracy of 82.3%. DeLong comparisons indicated that the combined signature outperformed each individual biomarker model (all *p* < 0.05). Table 6 presents the full ROC results, including PPV, NPV, and accuracy; the receiver operating characteristic curves for all four models are displayed in Figure 3A, and the comparative performance profile is summarized in Figures 3C,D.

**Table 6:** Exploratory predictive performance of single biomarkers and the combined serum signature.

Model	Optimal cutoff	AUC (95% CI)	Sensitivity, %	Specificity, %	PPV, %	NPV, %	Accuracy, %	Youden index
ENA-78	132.00 pg/mL	0.756 (0.692–0.812)	67.61	77.54	60.8	82.3	74.2	0.451
CXCL1	164.64 pg/mL	0.751 (0.687–0.808)	60.56	85.51	68.3	80.8	77.0	0.461
Caspase-3	7.10 ng/mL	0.777 (0.714–0.831)	63.04	84.51	67.7	81.6	77.2	0.476
Combined serum signature	Composite probability	0.893 (0.843–0.932)	83.10	81.88	70.2	90.4	82.3	0.650

PPV, NPV, and accuracy were calculated using the observed unfavorable early recovery prevalence of 34.0%. The combined serum signature had a significantly higher AUC than each individual biomarker by DeLong testing (*p* < 0.05).

### Internal validation and incremental value of the prediction model

**Figure 4:**
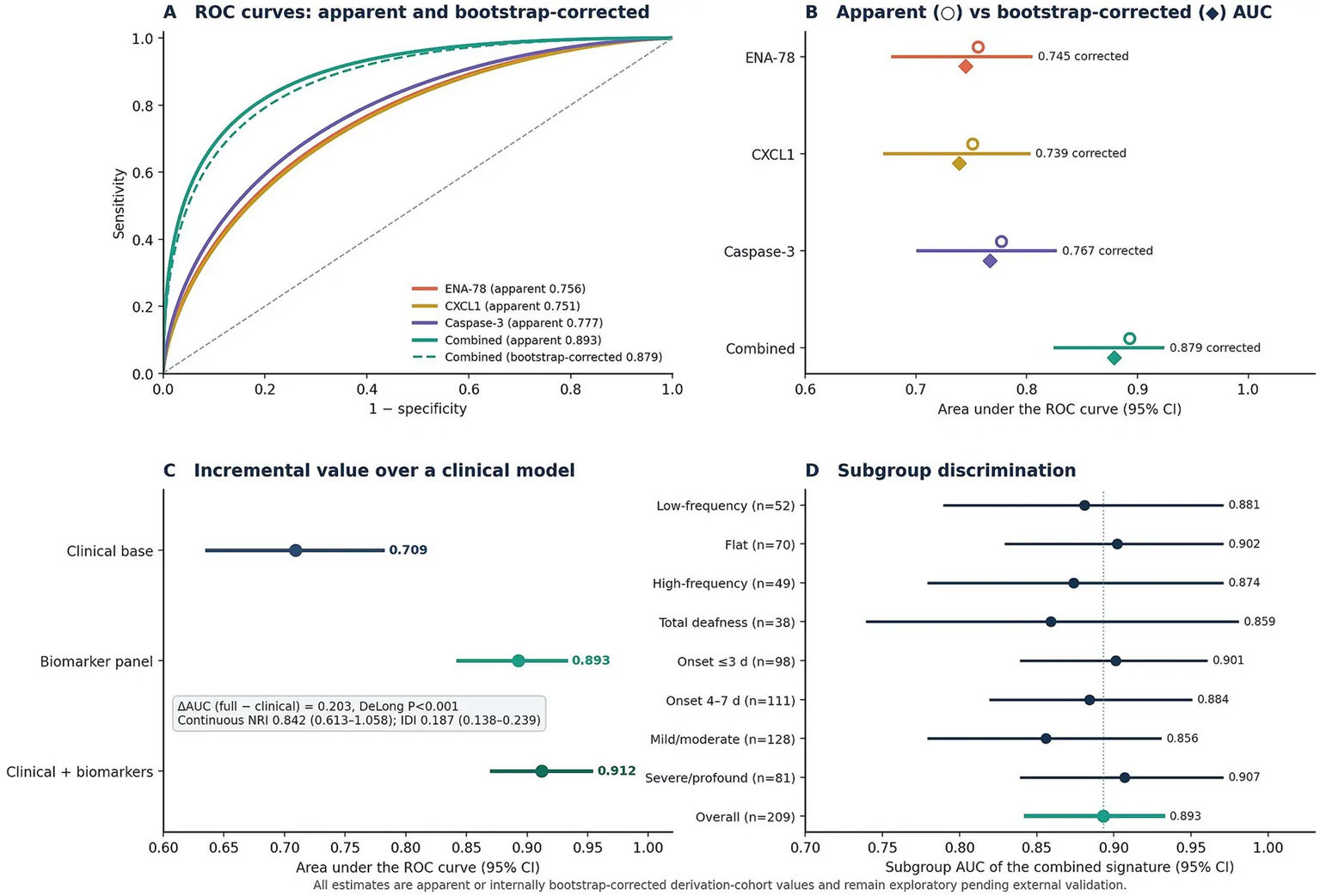
Internal validation, incremental value, and subgroup stability of the serum biomarker panel. **(A)** Shows the apparent receiver operating characteristic curves for the single markers and the combined signature together with the bootstrap-corrected curve for the combined model. **(B)** Compares apparent and 1,000-sample bootstrap-corrected areas under the curve for each model. **(C)** Displays the areas under the curve for the clinical base model, the biomarker panel, and the combined clinical-plus-biomarker model, with the change in AUC, the net reclassification improvement, and the integrated discrimination improvement annotated. Panel **(D)** is a forest plot of the discrimination of the combined signature within audiogram, treatment-delay, and severity subgroups, referenced to the overall value. All estimates are apparent or internally bootstrap-corrected derivation-cohort values and remain exploratory pending external validation.

Because the performance estimates described above are apparent values, 1,000-sample bootstrap resampling was used to quantify optimism, and the correction proved modest for every model. The optimism-corrected AUC was 0.745 for ENA-78, 0.739 for CXCL1, and 0.767 for caspase-3, and the combined serum signature retained strong discrimination after correction, with an optimism-corrected AUC of 0.879 (apparent AUC, 0.893; optimism, 0.014). Bootstrap-corrected sensitivity and specificity for the combined signature were 81.9 and 80.5%, respectively. The full internal-validation results are presented in Table 7 and Figures 4A,B. The uncorrected and bootstrap-corrected estimates are compared side by side in Supplementary Table S1.

**Table 7:** Internal (1,000-sample bootstrap) validation of the biomarker models.

Model	Apparent AUC	Optimism	Bootstrap-corrected AUC (95% CI)	Corrected sensitivity, %	Corrected specificity, %
ENA-78	0.756	0.011	0.745 (0.679–0.804)	64.8	76.1
CXCL1	0.751	0.012	0.739 (0.672–0.802)	58.9	84.0
Caspase-3	0.777	0.010	0.767 (0.702–0.826)	61.5	83.1
Combined serum signature	0.893	0.014	0.879 (0.826–0.923)	81.9	80.5

To determine whether the biomarkers added information beyond routinely available clinical variables, the clinical base model was compared with the combined clinical-plus-biomarker model. The clinical base model discriminated early recovery only moderately (apparent AUC, 0.709; 95% CI, 0.636–0.781). Adding serum ENA-78, CXCL1, and caspase-3 raised the AUC to 0.912 (95% CI, 0.871–0.953), an increase of 0.203 that was significant on DeLong testing (*p* < 0.001). The reclassification metrics reinforced this gain, with a continuous NRI of 0.842 (95% CI, 0.613–1.058) and an IDI of 0.187 (95% CI, 0.138–0.239; *p* < 0.001). The three inflammatory-apoptotic biomarkers therefore carried prognostic information that was not captured by baseline clinical and audiometric characteristics. The comparison is summarized in Table 8 and Figure 4C. The variables entered into the clinical base model are listed in Supplementary Table S2.

**Table 8:** Incremental value of the serum biomarkers over a clinical base model.

Model	AUC (95% CI)	ΔAUC vs base	DeLong P	NRI (95% CI)	IDI (95% CI)
Clinical base model	0.709 (0.636–0.781)	Reference	—	—	—
Biomarker panel	0.893 (0.843–0.932)	0.184	<0.001	—	—
Clinical + biomarkers	0.912 (0.871–0.953)	0.203	<0.001	0.842 (0.613–1.058)	0.187 (0.138–0.239)

**Figure 5:**
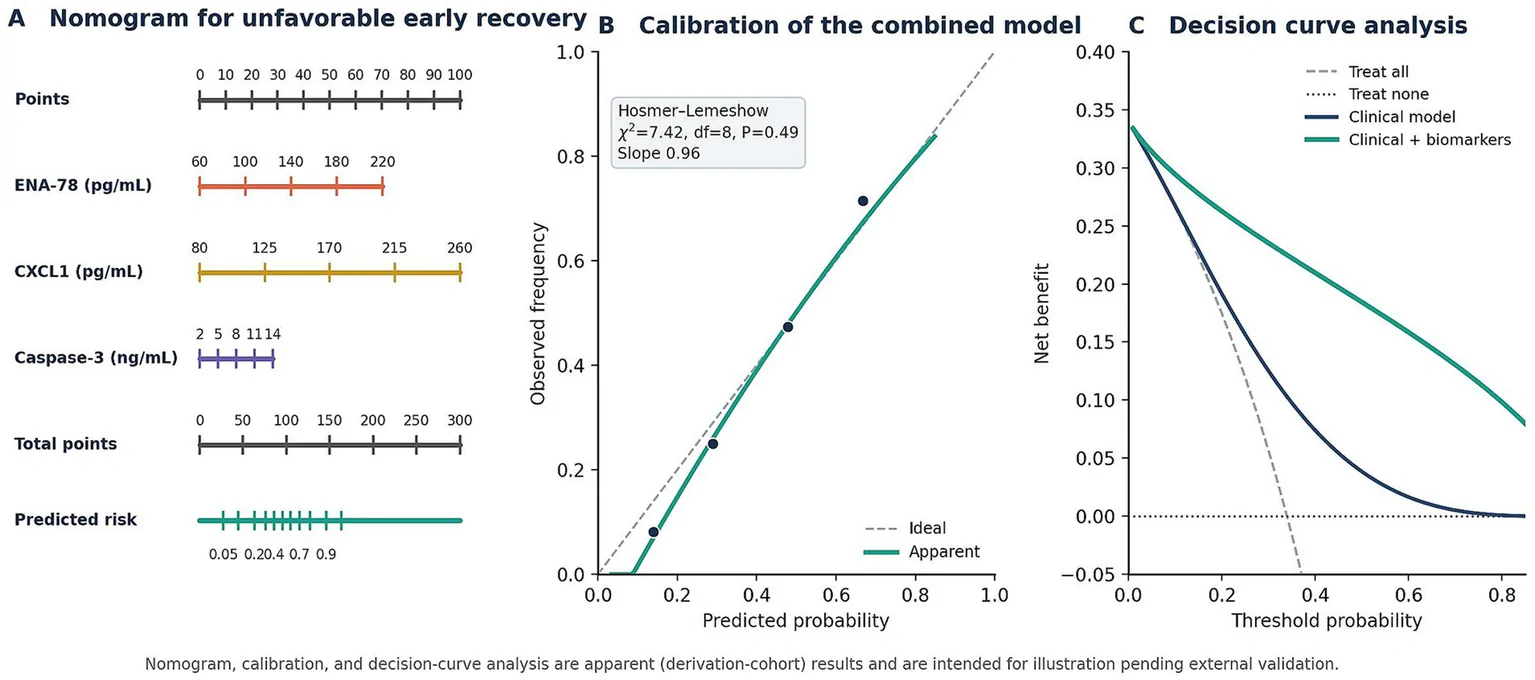
Clinical translation of the combined serum biomarker model. **(A)** Presents a nomogram derived from the combined model, in which admission ENA-78, CXCL1, and caspase-3 values are converted to points that sum to an individual predicted probability of unfavorable early recovery. **(B)** Shows the calibration curve relating predicted probabilities to observed early-recovery frequencies, with the Hosmer-Lemeshow statistic and calibration slope annotated. **(C)** Shows the decision-curve analysis, comparing the net benefit of the combined clinical-plus-biomarker model, the clinical base model, and the treat-all and treat-none strategies across threshold probabilities. All estimates are apparent derivation-cohort values and are intended for illustration pending external validation.

The combined model was well calibrated, with a nonsignificant Hosmer-Lemeshow test (χ^2^ = 7.42; df = 8; *p* = 0.49) and a calibration slope of 0.96, indicating close agreement between predicted probabilities and observed early-recovery frequencies (Figure 5B). A nomogram derived from the model translated the admission biomarker values into an individual predicted probability of unfavorable early recovery (Figure 5A). Decision-curve analysis showed that the combined model produced a higher net benefit than the clinical base model and than the treat-all and treat-none strategies across threshold probabilities of approximately 0.12 to 0.72, supporting its potential usefulness for admission-level risk stratification (Figure 5C). The calibration table by decile of predicted risk and the net benefit at representative thresholds are given in Supplementary Tables S4, S5, and the point allocation for the nomogram is tabulated in Supplementary Table S6.

### Subgroup analyses

The discriminative performance of the combined signature was examined within prespecified clinical subgroups. Discrimination remained consistent across audiogram configurations, with subgroup AUCs of 0.881 for the low-frequency type, 0.902 for the flat type, 0.874 for the high-frequency type, and 0.859 for total deafness. Performance was likewise stable when patients were stratified by treatment delay (AUC, 0.901 for admission within 3 days and 0.884 for admission at 4 to 7 days) and by baseline severity (AUC, 0.856 for mild or moderate loss and 0.907 for severe or profound loss). Every subgroup estimate overlapped the overall value of 0.893, indicating that the panel performed comparably across the clinical spectrum rather than being driven by any single phenotype. Subgroup results are detailed in Table 9 and Figure 4D. The corresponding areas under the curve for each single biomarker within every stratum are reported in Supplementary Table S3. Vestibular vertigo and tinnitus severity were not recorded uniformly in this cohort and could not be used as stratifying variables, as noted in the limitations.

**Table 9:** Discrimination of the combined serum signature within prespecified subgroups.

Subgroup	*n*	Events, *n*	AUC (95% CI)
Audiogram, low-frequency type	52	17	0.881 (0.79–0.97)
Audiogram, flat type	70	17	0.902 (0.83–0.97)
Audiogram, high-frequency type	49	22	0.874 (0.78–0.97)
Audiogram, total deafness	38	15	0.859 (0.74–0.98)
Onset-to-admission ≤3 days	98	27	0.901 (0.84–0.96)
Onset-to-admission 4–7 days	111	44	0.884 (0.82–0.95)
Baseline severity, mild or moderate	128	13	0.856 (0.78–0.93)
Baseline severity, severe or profound	81	58	0.907 (0.84–0.97)
Overall cohort	209	71	0.893 (0.843–0.932)

### Sensitivity analysis in the control cohort

To confirm that the case–control differences were not an artifact of unmeasured inflammatory comorbidity among controls, the control cohort was re-examined after the stricter exclusion criteria were applied and was stratified by any history of inflammatory conditions. Baseline biomarker levels did not differ meaningfully between controls with and without such a history for ENA-78 (63.40 ± 5.45 vs. 62.96 ± 5.42 pg/mL; *p* = 0.73), CXCL1 (76.43 ± 7.45 vs. 75.45 ± 6.70 pg/mL; *p* = 0.54), or caspase-3 (3.09 ± 1.00 vs. 3.38 ± 1.14 ng/mL; *p* = 0.28). The elevation observed in patients with ISSNHL therefore greatly exceeded any variation attributable to control inflammatory background (Table 10).

**Table 10:** Serum biomarker levels in controls stratified by history of inflammatory conditions (sensitivity analysis).

Biomarker	Inflammatory history (*n* = 20)	No inflammatory history (*n* = 189)	*p* value
ENA-78, pg/mL	63.40 ± 5.45	62.96 ± 5.42	0.73
CXCL1, pg/mL	76.43 ± 7.45	75.45 ± 6.70	0.54
Caspase-3, ng/mL	3.09 ± 1.00	3.38 ± 1.14	0.28

## Discussion

In this study, serum ENA-78, CXCL1, and caspase-3 were all elevated in patients with ISSNHL compared with healthy controls, increased progressively with worsening initial hearing-loss severity, and were significantly higher in patients who experienced unfavorable early hearing recovery after 10 days of standardized treatment. When the three biomarkers were entered simultaneously into a logistic regression model, each retained an independent association with outcome, and the combined serum signature demonstrated substantially stronger apparent discriminative performance than any single biomarker alone. Taken together, these findings support the concept that an inflammation-apoptosis biomarker axis may capture clinically relevant biological heterogeneity in ISSNHL.

The relationship between the biomarkers and initial severity is particularly noteworthy. ENA-78/CXCL5 and CXCL1 are chemokines with well-established neutrophil-attracting properties and recognized roles in inflammatory amplification, endothelial activation, and tissue injury in ischemic and inflammatory disease models (Xiao et al., 2022; Huang et al., 2023; Qiao et al., 2024). The cochlea is especially vulnerable to this type of insult because it depends on terminal circulation and has limited tolerance for reductions in oxygen delivery or microvascular patency (Lee and Chung, 2024; Tsuzuki and Wasano, 2024; Mandavia et al., 2024; Wu et al., 2023). A stepwise increase in ENA-78 and CXCL1 from mild to profound ISSNHL therefore fits a biologically plausible framework in which greater systemic chemokine activation mirrors a more intense cochlear inflammatory-microvascular insult. These findings also extend the growing ISSNHL literature on inflammatory biomarkers, which has generally focused on routine hematologic indices such as the neutrophil-to-lymphocyte ratio, platelet-to-lymphocyte ratio, or composite immune-inflammatory scores (Zhao et al., 2025; Zhang X. et al., 2025; Zhang Q. et al., 2025). By contrast, the present study emphasizes more specific chemotactic mediators that may sit closer to the underlying inflammatory biology. Prior reports have measured circulating endothelial and vascular mediators such as endothelin-1 and soluble vascular cell adhesion molecule-1 in sudden deafness (Wang and Zhang, 2024), and a small number of studies have detected elevated serum ENA-78 in affected patients (Al-Azzawi and Stapleton, 2023); however, these markers have generally been examined in isolation rather than as an integrated inflammation-apoptosis signature linked to both severity and early recovery.

Placing these markers alongside earlier work clarifies what the present panel adds. Studies of routine hematologic indices, such as the neutrophil-to-lymphocyte ratio and the systemic immune-inflammation index, have linked a general inflammatory state to ISSNHL occurrence and outcome, yet such indices are biologically nonspecific and cannot separate inflammatory recruitment from tissue injury. Reports that examined ENA-78 or vascular adhesion molecules in sudden deafness have generally measured a single mediator and related it to disease presence rather than to graded severity or treatment response, and the experimental evidence for caspase-dependent cochlear cell death has remained largely preclinical. By measuring an upstream chemokine pair together with a downstream apoptotic effector in the same patients, and by relating them at once to severity and to early recovery, the present study draws these previously separate lines of evidence into a single, clinically anchored signature.

Caspase-3 showed the largest odds ratio in the multivariable model and the highest AUC among the individual biomarkers. This observation is clinically meaningful because caspase-3 is a terminal executioner protease within the apoptotic cascade. Experimental studies of the inner ear have shown that inflammatory and oxidative stress can activate programmed cell death in cochlear hair cells and supporting structures, and may thereby be associated with reduced reversibility of auditory dysfunction (Qiao et al., 2024; Yu et al., 2022; Wu et al., 2025). It is therefore plausible that higher serum caspase-3 reflects a more advanced injury state in which reversible inflammatory dysfunction has begun to transition toward irreversible cellular damage. Nevertheless, the interpretation must remain appropriately cautious. Serum caspase-3 does not directly localize apoptosis to the cochlea and should not be described as a definitive measure of hair-cell death. Rather, it is better understood as an accessible systemic correlate of apoptosis-associated injury burden that may track with the severity and reversibility of the auditory insult.

The most compelling analytic finding was the performance of the combined serum signature. Although ENA-78, CXCL1, and caspase-3 each showed moderate apparent discrimination when evaluated alone, their combination yielded an AUC of 0.893 with balanced sensitivity and specificity. This pattern suggests that the chemokine-related and apoptosis-related signals are complementary rather than redundant. ENA-78 and CXCL1 likely reflect upstream inflammatory recruitment, endothelial activation, and microvascular stress, whereas caspase-3 reflects a downstream injury phase in which irreversible cellular damage may be more likely. A combined panel therefore appears to capture a broader injury continuum than a single biomarker. This interpretation is consistent with the broader direction of contemporary ISSNHL prognostic research, which increasingly emphasizes integrated clinical prediction rather than reliance on isolated indicators (Mandavia et al., 2024; Wu et al., 2023; Zhang et al., 2023; Shon et al., 2024).

Several analyses were added to clarify the clinical meaning of the combined signature rather than to rely on discrimination alone. After 1,000-sample bootstrap correction the combined AUC fell only slightly, from 0.893 to 0.879, which suggests that the apparent performance was not substantially inflated by overfitting in a cohort with 71 outcome events and three predictors. More important, the biomarkers added clear information beyond routine clinical assessment. A model built from age, sex, vascular comorbidity, treatment delay, baseline PTA, and audiogram configuration discriminated early recovery only moderately, whereas adding the three serum markers raised the AUC by more than 0.20 and produced a large, statistically significant NRI and IDI. This incremental value is the central argument for measuring the panel, because a biomarker that merely echoes information already available at the bedside would add little.

The nomogram, calibration curve, and decision-curve analysis translate these statistical gains into a form clinicians can use. The well-calibrated nomogram allows an individual admission risk to be read directly from the three biomarker values, and the decision-curve analysis indicates that acting on this risk provides net benefit across a broad range of thresholds rather than at a single arbitrary cutoff. The stability of discrimination across audiogram, treatment-delay, and severity subgroups further suggests that the panel reflects a shared biological process rather than a phenotype-specific epiphenomenon. These findings should nonetheless be regarded as internally validated and exploratory, because all estimates were derived from a single derivation cohort and were not tested in an independent population.

From a clinical standpoint, these biomarkers may have value as adjunctive tools for early risk stratification. Because the outcome heterogeneity of ISSNHL remains difficult to predict using routine assessment alone, a readily measurable serum panel that identifies patients at increased risk of poor early recovery could improve counseling and follow-up planning. For example, patients with a heavier inflammatory-apoptotic biomarker burden at presentation might warrant more intensive early surveillance, closer audiometric reassessment, or earlier consideration of escalation or salvage approaches within institutional practice patterns. At the same time, the current findings should not be overinterpreted. The panel has not yet been shown to provide incremental benefit over a comprehensive clinical model incorporating variables such as vertigo, tinnitus severity, baseline speech discrimination, or long-term hearing outcomes. Its role at present is therefore hypothesis-generating and potentially translational rather than immediately practice-changing.

This study also has several strengths that support the reliability of its findings. Patients were enrolled consecutively over a defined period, which limits selection bias, and all received an identical, guideline-based 10-day regimen, so that outcome differences are unlikely to reflect variation in treatment. Serum markers were measured in duplicate under blinded, standardized laboratory conditions with low assay variability, and posttreatment audiometry was performed by an assessor unaware of both group allocation and biomarker results. The sample was relatively large for a single-center biomarker study, and the analysis was reinforced by bootstrap internal validation, formal comparison with a clinical model, calibration and decision-curve assessment, and prespecified subgroup analyses.

Several limitations must also be acknowledged. First, this was a single-center observational study, and the results may therefore be influenced by local referral patterns, treatment practices, or patient characteristics; external validity beyond this setting remains to be established. Second, the endpoint was early hearing recovery after 10 days of treatment, which should not be equated with definitive long-term prognosis. Recovery at 1 month, 3 months, and 6 months would provide a more robust and clinically durable endpoint. Third, the prediction model was developed and internally validated in a single derivation cohort. Bootstrap resampling reduced the apparent AUC only slightly and calibration was adequate, but no external cohort was available, so residual optimism cannot be excluded and transportability to other centers and populations remains unproven. Fourth, the clinical base model was restricted to the variables recorded in this cohort, and established prognostic factors such as vestibular vertigo, tinnitus severity, and speech-discrimination scores were not captured, so the incremental value reported here may change once they are included; the present results should accordingly be read as exploratory biomarker associations rather than a finalized clinical prediction model. Finally, serum biomarkers cannot directly characterize the cochlear microenvironment, and the observational design does not permit causal inference regarding the mechanistic sequence linking chemokine activation, apoptosis, and treatment response. The associations reported here are therefore best understood as systemic correlates of an underlying inflammatory and apoptotic process rather than as evidence of direct cochlear injury.

Future work should therefore prioritize external validation in independent multicenter cohorts, prospective collection of the full range of clinical prognostic variables, extension of the endpoint to long-term audiological follow-up, and complementary cellular and animal studies to test whether the inflammatory-apoptotic axis identified here is mechanistically involved in cochlear injury rather than merely associated with it.

In conclusion, admission serum ENA-78, CXCL1, and caspase-3 concentrations were closely associated with both initial disease severity and early hearing recovery in adults with ISSNHL. The combined serum signature demonstrated stronger apparent predictive performance than any individual biomarker, supporting the notion that a biologically integrated inflammation-apoptosis panel may have value for early risk stratification. Multicenter studies with longer follow-up, broader clinical covariate capture, and formal model validation will be necessary to determine whether this biomarker strategy can be translated into a clinically useful prognostic tool.

## Data Availability

The raw data supporting the conclusions of this article will be made available by the authors, without undue reservation.
