# Supplementary material for: Serum ENA-78, CXCL1, and caspase-3 are associated with disease severity and early hearing recovery in idiopathic sudden sensorineural hearing loss
Source: Front Neurosci. 2026 Aug 20;20:1901582. doi: 10.3389/fnins.2026.1901582 (PMC13538873; doi:10.3389/fnins.2026.1901582)
Supplement: Supplementary file 1 [file Table_1.docx]

**Supplementary Material**

*Serum ENA-78, CXCL1, and Caspase-3 Are Associated with Disease Severity and Early Hearing Recovery in Idiopathic Sudden Sensorineural Hearing Loss*

This file contains the supplementary statistical analyses that support the prediction-model results reported in the main manuscript. Supplementary Table S1 presents the uncorrected and bootstrap-corrected performance of every model side by side. Table S2 gives the composition of the clinical base model used for the incremental-value comparison. Table S3 extends the subgroup analysis to each single biomarker as well as the combined signature. Tables S4 and S5 provide the numerical detail behind the calibration curve and the decision curve shown in Figure 4. Table S6 allows the nomogram to be applied without reference to the figure.

All estimates are apparent or internally bootstrap-corrected values derived from the single development cohort of 209 patients with 71 unfavorable early-recovery events. They have not been validated externally and should be interpreted as exploratory.

**Supplementary Table S1. Uncorrected and 1000-sample bootstrap-corrected performance of each model.**

| **Model** | **Optimal cutoff** | **Apparent AUC (95% CI)** | **Apparent sensitivity / specificity, %** | **Optimism** | **Bootstrap-corrected AUC (95% CI)** | **Corrected sensitivity / specificity, %** |
| --- | --- | --- | --- | --- | --- | --- |
| ENA-78 | 132.00 pg/mL | 0.756 (0.692-0.812) | 67.61 / 77.54 | 0.011 | 0.745 (0.679-0.804) | 64.8 / 76.1 |
| CXCL1 | 164.64 pg/mL | 0.751 (0.687-0.808) | 60.56 / 85.51 | 0.012 | 0.739 (0.672-0.802) | 58.9 / 84.0 |
| Caspase-3 | 7.10 ng/mL | 0.777 (0.714-0.831) | 63.04 / 84.51 | 0.010 | 0.767 (0.702-0.826) | 61.5 / 83.1 |
| Combined serum signature | Predicted risk 0.34 | 0.893 (0.843-0.932) | 83.10 / 81.88 | 0.014 | 0.879 (0.826-0.923) | 81.9 / 80.5 |

*Optimism is the mean difference between the apparent AUC and the AUC obtained in the bootstrap samples. The corrected AUC is the apparent AUC minus the optimism. Cutoffs were selected by maximizing the Youden index.*

**Supplementary Table S2. Composition of the clinical base model for unfavorable early hearing recovery.**

| **Variable** | **Odds ratio** | **95% CI** | **P value** |
| --- | --- | --- | --- |
| Baseline PTA, per 10 dB | 1.62 | 1.20-2.20 | 0.002 |
| Audiogram, total deafness (vs flat type) | 1.71 | 0.90-3.27 | 0.104 |
| Audiogram, high-frequency (vs flat type) | 1.18 | 0.64-2.19 | 0.599 |
| Audiogram, low-frequency (vs flat type) | 0.82 | 0.43-1.57 | 0.548 |
| Onset-to-admission, per day | 1.14 | 0.87-1.49 | 0.332 |
| Age, per year | 1.01 | 0.95-1.07 | 0.748 |
| Male sex | 1.06 | 0.58-1.95 | 0.851 |
| BMI, per kg/m² | 1.03 | 0.85-1.25 | 0.763 |
| Diabetes mellitus | 1.32 | 0.43-4.03 | 0.626 |
| Hypertension | 1.21 | 0.52-2.81 | 0.658 |
| Hyperlipidemia | 1.11 | 0.42-2.90 | 0.831 |

*The clinical base model contained only variables that were available at admission for the whole cohort. Its apparent AUC was 0.709 (95% CI, 0.636-0.781). Vestibular vertigo, tinnitus severity, and speech-discrimination scores were not recorded uniformly and could not be entered, as stated in the limitations.*

**Supplementary Table S3. Discrimination of each single biomarker and of the combined signature within prespecified subgroups.**

| **Subgroup** | **n** | **Events, n** | **ENA-78, AUC** | **CXCL1, AUC** | **Caspase-3, AUC** | **Combined, AUC** |
| --- | --- | --- | --- | --- | --- | --- |
| Audiogram, low-frequency type | 52 | 17 | 0.743 | 0.729 | 0.735 | 0.881 |
| Audiogram, flat type | 70 | 17 | 0.776 | 0.766 | 0.791 | 0.902 |
| Audiogram, high-frequency type | 49 | 22 | 0.756 | 0.704 | 0.747 | 0.874 |
| Audiogram, total deafness | 38 | 15 | 0.698 | 0.740 | 0.745 | 0.859 |
| Onset-to-admission ≤3 days | 98 | 27 | 0.762 | 0.752 | 0.799 | 0.901 |
| Onset-to-admission 4–7 days | 111 | 44 | 0.722 | 0.770 | 0.757 | 0.884 |
| Baseline severity, mild or moderate | 128 | 13 | 0.734 | 0.689 | 0.764 | 0.856 |
| Baseline severity, severe or profound | 81 | 58 | 0.780 | 0.775 | 0.808 | 0.907 |
| Overall cohort | 209 | 71 | 0.756 | 0.751 | 0.777 | 0.893 |

*Subgroup sizes, event counts, and the combined-signature values correspond to main-text Table 9. Within every stratum the combined signature discriminated better than any single biomarker, and the ordering of the three individual markers was preserved.*

**Supplementary Table S4. Calibration of the combined model by decile of predicted risk.**

| **Decile** | **Predicted risk range** | **n** | **Observed events** | **Expected events** | **Observed, %** | **Expected, %** |
| --- | --- | --- | --- | --- | --- | --- |
| 1 | 0.00-0.01 | 21 | 0 | 0.1 | 0.0 | 0.6 |
| 2 | 0.01-0.03 | 21 | 0 | 0.4 | 0.0 | 1.9 |
| 3 | 0.03-0.06 | 21 | 2 | 0.9 | 9.5 | 4.4 |
| 4 | 0.06-0.10 | 21 | 2 | 1.6 | 9.5 | 7.7 |
| 5 | 0.10-0.15 | 21 | 4 | 2.7 | 19.0 | 12.9 |
| 6 | 0.15-0.33 | 21 | 2 | 4.8 | 9.5 | 23.0 |
| 7 | 0.34-0.55 | 21 | 7 | 9.2 | 33.3 | 43.8 |
| 8 | 0.55-0.78 | 21 | 16 | 14.2 | 76.2 | 67.6 |
| 9 | 0.80-0.90 | 21 | 18 | 17.9 | 85.7 | 85.3 |
| 10 | 0.92-0.98 | 20 | 20 | 19.1 | 100.0 | 95.5 |

*Hosmer-Lemeshow χ² = 7.42, df = 8, P = 0.49; calibration slope 0.96. Observed and expected event counts agree closely across the full range of predicted risk, which corresponds to the calibration curve in Figure 4B.*

**Supplementary Table S5. Net benefit at representative threshold probabilities (decision-curve analysis).**

| **Threshold probability** | **Clinical + biomarker model** | **Clinical base model** | **Treat all** | **Treat none** |
| --- | --- | --- | --- | --- |
| 0.10 | 0.2924 | 0.2701 | 0.2663 | 0.0000 |
| 0.15 | 0.2868 | 0.2291 | 0.2232 | 0.0000 |
| 0.20 | 0.2751 | 0.1914 | 0.1746 | 0.0000 |
| 0.30 | 0.2481 | 0.1422 | 0.0567 | 0.0000 |
| 0.40 | 0.2233 | 0.0718 | -0.1005 | 0.0000 |
| 0.50 | 0.2010 | 0.0431 | -0.3206 | 0.0000 |
| 0.60 | 0.1746 | 0.0167 | -0.6507 | 0.0000 |
| 0.70 | 0.1324 | -0.0064 | -1.2010 | 0.0000 |
| 0.75 | 0.1292 | -0.0191 | -1.6411 | 0.0000 |

*Net benefit was calculated as (true positives / n) − (false positives / n) × [threshold / (1 − threshold)]. The combined model gave the highest net benefit at every threshold examined, and remained above the treat-all and treat-none strategies across approximately 0.12 to 0.72, as shown in Figure 4C.*

**Supplementary Table S6. Point allocation for the nomogram and conversion of total points to predicted risk.**

| **Predictor** | **Value → points** | **Value → points** | **Value → points** | **Value → points** | **Value → points** |
| --- | --- | --- | --- | --- | --- |
| ENA-78, pg/mL | 60 → 0 | 100 → 18 | 140 → 35 | 180 → 53 | 220 → 70 |
| CXCL1, pg/mL | 80 → 0 | 125 → 25 | 170 → 50 | 215 → 75 | 260 → 100 |
| Caspase-3, ng/mL | 2 → 0 | 5 → 7 | 8 → 14 | 11 → 21 | 14 → 28 |

**Conversion of total points to predicted probability of unfavorable early recovery**

| **Total points** | **Predicted risk** |
| --- | --- |
| 0 | 0.016 |
| 50 | 0.123 |
| 100 | 0.548 |
| 150 | 0.913 |
| 200 | 0.989 |
| 250 | 0.999 |
| 300 | 1.000 |

*Points are assigned by linear interpolation between the tabulated values. The model underlying the nomogram is logit(p) = −7.39 + 0.019 × ENA-78 + 0.024 × CXCL1 + 0.101 × caspase-3, with ENA-78 and CXCL1 in pg/mL and caspase-3 in ng/mL.*
